# A Sorting Statistic with Application in Neurological Magnetic Resonance Imaging of Autism

**DOI:** 10.1155/2018/8039075

**Published:** 2018-03-29

**Authors:** Jacob Levman, Emi Takahashi, Cynthia Forgeron, Patrick MacDonald, Natalie Stewart, Ashley Lim, Anne Martel

**Affiliations:** ^1^Department of Mathematics, Statistics and Computer Science, St. Francis Xavier University, Antigonish, NS, Canada B2G 2W5; ^2^Division of Newborn Medicine, Department of Medicine, Boston Children's Hospital, Harvard Medical School, 1 Autumn Street, No. 456, Boston, MA 02215, USA; ^3^Athinoula A. Martinos Center for Biomedical Imaging, Massachusetts General Hospital, Harvard Medical School, 149 13th Street, Charlestown, MA 02129, USA; ^4^Sunnybrook Research Institute, Sunnybrook Health Sciences Centre, Department of Medical Biophysics, University of Toronto, Toronto, ON, Canada

## Abstract

Effect size refers to the assessment of the extent of differences between two groups of samples on a single measurement. Assessing effect size in medical research is typically accomplished with Cohen's *d* statistic. Cohen's *d* statistic assumes that average values are good estimators of the position of a distribution of numbers and also assumes Gaussian (or bell-shaped) underlying data distributions. In this paper, we present an alternative evaluative statistic that can quantify differences between two data distributions in a manner that is similar to traditional effect size calculations; however, the proposed approach avoids making assumptions regarding the shape of the underlying data distribution. The proposed sorting statistic is compared with Cohen's *d* statistic and is demonstrated to be capable of identifying feature measurements of potential interest for which Cohen's *d* statistic implies the measurement would be of little use. This proposed sorting statistic has been evaluated on a large clinical autism dataset from *Boston Children's Hospital*, *Harvard Medical School*, demonstrating that it can potentially play a constructive role in future healthcare technologies.

## 1. Introduction

Cohen's *d* statistic was introduced to address a shortage of appropriate statistical metrics for use in the behavioral sciences [1], where it was shown that the *d* statistic is capable of quantifying a range of small, medium, and large effect sizes representing the extent of group-wise differences observed in a given experiment. While the approach simplified comparing results in psychological/behavioral analysis and is a strong indicator of statistical significance, it is not without shortcomings. The *d* statistic assumes the underlying data distribution is bell-shaped or Gaussian [2] and the average (or mean) positional markers of the distributions are known to drift in the presence of outliers and when the underlying distributions are skewed. When applying Cohen's *d* statistic, discrepancies between the assumed bell-shaped distribution and the actual distribution can lead to erroneous findings. Demonstrating an effect where one is not present in the underlying data or showing no effect when group-wise differences are present can lead to inaccurate analyses.

Statistics based on sorting or ranking measurements have been proposed previously, such as the Wilcoxon rank sum method, a nonparametric test [3]. The Wilcoxon rank sum test acts as an alternative to Student's *t*-test [4], which are methods used for computing probabilities (*p* values) and performing hypothesis testing, not for assessing effect size or group-wise differences between two distributions of measured values. The Wilcoxon rank sum test does not assume the underlying distribution is normal or bell-shaped, providing a major advantage over Student's *t*-test which assumes the existence of an underlying Gaussian distribution. This is a significant advantage because it reduces the possibility of producing erroneous findings while remaining nearly as efficient as the *t*-test [5]. However, the Wilcoxon rank sum method is a hypothesis test and not an effect size estimator similar to Cohen's *d*. In this paper, we present a nonparametric statistic that can assess group-wise differences between two distributions producing similar results to Cohen's *d* statistic while avoiding making assumptions about the underlying shape of the distribution of the data by employing sorting statistics, similar to what was accomplished with the Wilcoxon sign-rank test.

This article demonstrates the use of statistical analysis metrics on an autism dataset from *Boston Children's Hospital* (BCH), *Harvard Medical School*. Autism is characterized by repetitive, stereotyped behavior, impaired social communication, and deficits in social reciprocity [6, 7]. Evidence of neuroanatomical differences between autistic patients and healthy controls comes from postmortem and neuroimaging studies [8, 9]. Magnetic resonance imaging (MRI) provides physiological and anatomical measurements of a patient's brain, information that has the potential to assist in healthcare technologies and basic research. The most commonly used MRI method provides clinically useful soft tissue contrast. In the brain, MRI can differentiate between gray matter, white matter, and cerebrospinal fluid, which forms the basis for automated pattern recognition technology, which extracts measurements such as white matter volumes, cortical thicknesses, cortical curvature and measurements [10]. The analysis of autistic patients who have undergone MRI examinations has been the subject of many studies in the literature [11–25] that have incorporated distributed quantification of volumes, cortical thickness, surface areas, and so on [10].

Major structural changes occur between children and adults [26–32], making analysis of pediatric populations extra challenging. Distributed patterns of brain activity and structure provide important brain function information [33–36], and identifying these patterns is particularly challenging in a preadult population, because of a rapidly changing anatomy and physiology, small brain sizes, participant motion, a high degree of brain plasticity, and an incomplete understanding of brain development.

In this paper, we demonstrate a novel sorting statistic as a method for assisting in the analysis of group-wise differences in the neurological presentation of healthy and autistic children who received MRI examinations at BCH at 3 Tesla producing volumetric T1 examinations compatible with the automated extraction of distributed measurements [10].

## 2. Materials and Methods

### 2.1. Participants

Following approval by the Institutional Review Board at BCH, the clinical imaging electronic database at BCH was reviewed and participants for whom autism was indicated in their electronic medical records were included for further analysis. Examinations deemed to be of low quality (excessive participant motion etc.), and those that were inaccessible for technical reasons were excluded from the study, yielding 1003 examinations from 781 autistic participants. Healthy participants were assembled retrospectively in a previous analysis [37] by selecting individuals with a normal MRI examination assessed by a BCH neuroradiologist and whose medical records provided no indication of neurological problems. The exclusion criteria applied to the autistic population were also applied to the healthy participants. This yielded 993 examinations from 988 healthy participants for inclusion in this analysis. This population represents a demographic of participants imaged as part of routine clinical imaging (ages 0 to 32 years).

### 2.2. MRI Data Acquisition and Preprocessing

Participants were imaged with clinical 3 Tesla MRI scanners (Skyra, Siemens Medical Systems, Erlangen, Germany) at BCH. This produced T1 structural volumetric images accessed with the Children's Research and Integration System [38]. Motion correction was not performed; however, based on visual assessment, examinations with substantial motion artifacts were carefully excluded. T1 volumetric examinations were analyzed with FreeSurfer [10], which provides regions-of-interest across a participant's brain along with a variety of localized measurements therein. The extracted measurements are all structural and based on the T1 volumetric examination only. The results produced by FreeSurfer for each examination were displayed with label map overlays and visually inspected for quality of regional segmentation results. If results were observed to substantially fail, they were excluded.

### 2.3. Quantification and Statistical Analysis

This study included the acquisition of 4788 regionally distributed measurements per imaging examination, as extracted by FreeSurfer's recon-all command which processes the input examination with all available neuroanatomical brain atlases [10]. This provides a wide variety of measurements including regional volumes, cortical thicknesses, and surface curvature. For each acquired measurement, we compute Cohen's *d* statistic (1), an established method for assessing effect size and widely used in neuroimaging research to evaluate the amount of group-wise separation between two distributions of samples [1]. Our data includes instances of our measurements from 1003 autistic and 993 healthy participants.

(1) $$\begin{array}{c} d=\frac{\bar{x}-\bar{y}}{\sigma }, \end{array}$$

where the numerator represents the difference between the mean values of the two distributions *x* (autism) and *y* (healthy), and *σ* represents the standard deviation of the joint distribution. Cohen's *d* statistic assumes the underlying data follows a bell-shaped (or Gaussian) distribution, by employing the standard deviation spread measurement. Cohen's *d* statistic also assumes that average values are reliable measures of the location/position of a distribution; however, outliers can induce drift in these point estimators.

For each acquired measurement, we also compute our proposed sorting statistic which has properties similar to a traditional effect size calculation but does not make assumptions about the shape of the underlying distribution. The proposed sorting statistic is defined in

(2) $$\begin{array}{c} D_{s}=\frac{\tilde{x}-\tilde{y}}{\tilde{Z_{x}}-\tilde{Z_{y}}}, \end{array}$$

where *x* and *y* are the two input distributions for which $\tilde{x}>\tilde{y}.$$\tilde{x}$ is the median of the distribution *x* which has *n* samples. $\tilde{y}$ is the median of the distribution *y* which has *m* samples. Z is the joint distribution [*x*,*y*] sorted in descending order (highest to lowest). $\tilde{z_{x}}$ is the median of the first *n* samples of the sorted joint distribution Z. $\tilde{z_{y}}$ is the median of the final *m* samples of the sorted joint distribution Z.

The equation measures the simple distance between the positional estimates of each distribution (the difference between the medians), as the numerator. The denominator represents a theoretical maximum possible distance between the positional estimates of the two distributions, if we accept the number of measurements provided in each distribution as fixed and the values of those measurements as fixed but allow class membership to vary. This normalization procedure forces the resultant sorting statistic to take on values ranging from 0 to 1, with high values representing pairs of distributions that are more dissimilar to each other and values near 0 representing pairs of distributions that are nearly identical. Note that if the median of *x* is equal to the median of *y*, then (2) returns a value of zero.

A correlation analysis comparing the relationship between the proposed sorting statistic and the positively valued Cohen's *d* statistic was performed, along with a correlation analysis comparing the proposed sorting statistic with the negatively valued Cohen's *d* statistic. All statistical analyses were performed with Matlab (R2016a, Natick, MA, USA).

## 3. Results

**Figure 1:**
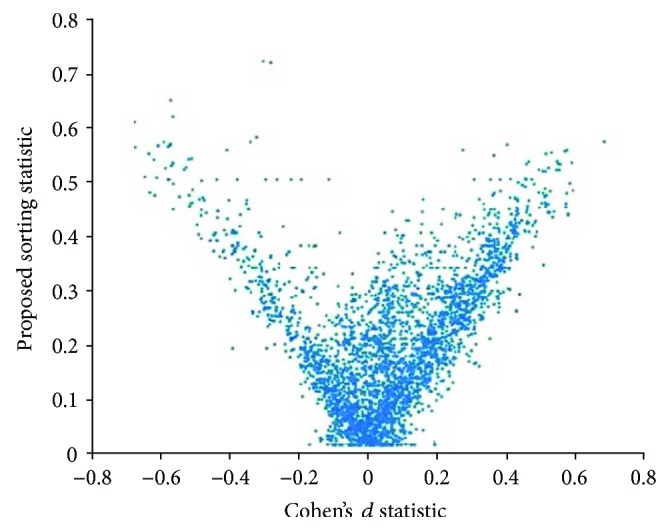
The relationship between the proposed sorting statistic and Cohen's *d* statistic for 4788 measurements extracted from healthy clinical participants and those with autism.

Each of the 4788 measurements acquired using FreeSurfer was compared on a group-wise basis (autistic versus healthy) using both Cohen's *d* statistic and the proposed sorting statistic. A plot of the relationship between Cohen's *d* statistic and the proposed sorting statistic across all measurements is provided in Figure 1.

**Figure 2:**
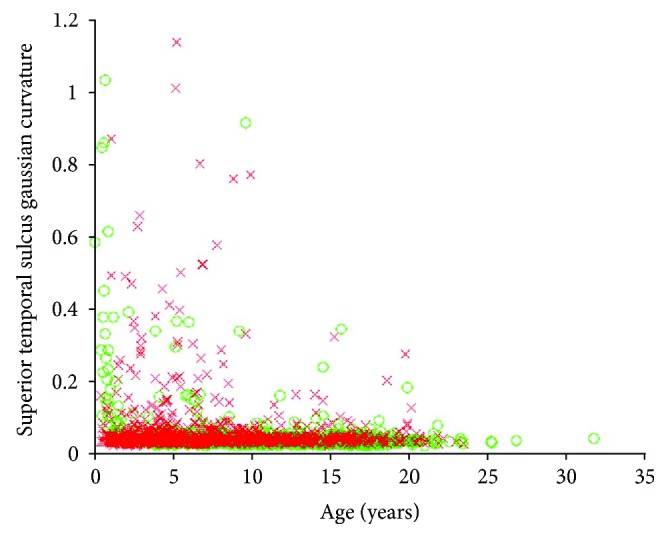
The Gaussian curvature of the surface of the superior temporal sulcus. Autistic participants are provided with a red x, healthy participants with a green o.

**Figure 3:**
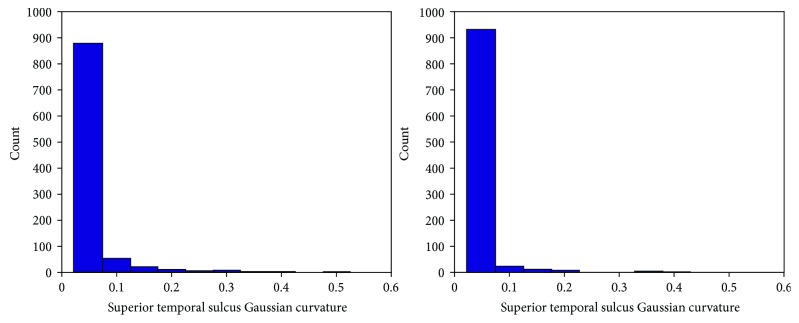
Histograms of the Gaussian curvature of the superior temporal sulcus in the autistic population (a) and the healthy population (b), demonstrating two naturally occurring skewed distributions in this dataset.

Note the strong positive correlation between the proposed sorting statistic and the positively valued Cohen's *d* statistic (rho = 0.7648, *p* = 0) as well as the strong negative correlation between the proposed sorting statistic and the negatively valued Cohen's *d* statistic (rho = −0.8314, *p* = 0). The strong correlations form a clear V-like pattern, the arms of the “V” representing a general agreement between the two measurements in assessing differences between the autistic and healthy populations, whereby increases in our proposed sorting statistic are strongly associated with Cohen's *d* statistic moving further away from zero. The region in between the two arms of the “V” (central regions of Figure 1) represents a region of data space of particular interest. This represents feature measurements for which the proposed sorting statistic implies that there might be more group-wise differences between the autistic and healthy groups than Cohen's *d* statistic implies. One such example is the Gaussian curvature of the surface of the superior temporal sulcus (Figure 2) whose Cohen's *d* statistic was merely 0.05, implying an uninteresting finding, but whose proposed sorting statistic yielded 0.4, implying that some separation between the two groups has been quantified. The two distributions (healthy and autistic) are highly overlapping and as you can see from their histograms (Figure 3); these are skewed distributions that deviate from normality. Skewed distributions are also known to cause drift on mean-based positional estimates which would affect Cohen's *d* statistic. Results indicate that values for the Gaussian curvature of the superior temporal sulcus (GCSTS) above about 0.1 may have potential in helping characterize autism as there are many more autistic samples with elevated GCSTS values relative to the healthy participants, despite the fact that low GCSTS values (~0.05) fall in a region of high overlap between our two groups of interest.

## 4. Discussion

Figure 1 demonstrates that the proposed sorting statistic is highly positively correlated with positive Cohen's *d* statistic (rho = 0.7648, *p* = 0), as well as highly negatively correlated with negative Cohen's *d* values (rho = −0.8314, *p* = 0). Measurements with an elevated sorting statistic and low Cohen's *d* (see central regions of Figure 1, raised from the bottom of the plot) represent measurements for which Cohen's *d* implies little to no effect, but for which the sorting statistic implies some separation of the two groups exists. The Gaussian curvature of the superior temporal sulcus (Figures 2 and 3) is one such example and can be found on Figure 1 at *d* = 0.05, *D*_s_ = 0.4. Additionally, it should be noted that the bottom left and bottom right of Figure 1 have no representative measurements falling in this zone. This indicates that there were no measurements in our dataset for which the proposed sorting statistic yielded a low value (implying the measurement is not useful), when Cohen's *d* statistic was producing large effect sizes. This implies that the proposed technique is not overlooking important feature measurements emphasized by Cohen's *d* statistic and so represents a desirable feature of the proposed sorting statistic.

Gaussian distributions are extremely common in real-world data analysis, which has supported the widespread use of Cohen's *d* statistic as a measurement for assessing group-wise differences and effect sizes. Unfortunately, naturally occurring data is also capable of deviating from normality, with Figures 2 and 3 demonstrating a naturally occurring data measurement that exhibits a skewed distribution. Additionally, it is known that in skewed distributions, the mean positional marker tends to drift towards the skewed tail end of the distribution, which may result in the average value being inadequate as a potential distribution positional marker. Figures 2 and 3 demonstrate an example where Cohen's *d* may mislead a reader towards interpreting a lack of group-wise differences between the autistic and healthy populations (*d* = 0.05). Although the distributions in Figures 2 and 3 include many sample measurements close to zero in either group, the autistic group clearly has more samples exhibiting elevated curvature values, causing an increase in the skew of the distribution relative to the healthy controls. This difference in skew is associated with a certain amount of potential from this variable to contribute to the characterization of autism, in particular, by potentially using this measurement as part of a multivariate machine learning technology.

The superior temporal region (Figures 2 and 3) is thought to be important in determining where others' emotions are being directed [39]; it includes the primary and part of the association auditory cortex [40], and it is thought to be involved in the perception of emotions in facial stimuli [41]. Thus, abnormalities of the superior temporal region may be associated with known autistic abnormalities of emotional processing, language processing, and visual/facial processing. Future work will focus on a detailed investigation of the curvature of the surface of a variety of neurological regions (including the superior temporal sulcus) as potential mechanisms for characterizing autism. Although the highlighted Gaussian curvature measurement only provides a modest amount of separation between our healthy and autistic populations, it does have some potential, which was not adequately captured by Cohen's *d* statistic because of assumptions made about the underlying data distribution. This helps demonstrate the proposed sorting statistical metric's potential to assist in the identification of feature measurements that may contribute to the accurate prediction of disease status in the context of a multivariate machine learning technology responsible for combining a series of measurements (each with some diagnostic potential) to form a final prediction. Future work will assess this proposed sorting statistic's potential to play a role in assessing an individual measurement's potential to contribute to multivariate machine learning technologies for health care.

Cohen suggested that a *d* statistic of 0.2 corresponds to a small effect size, 0.5 to a medium effect size, and 0.8 to a large effect size. Based on our autism data and referring to a visual inspection of Figure 1, we provide the roughly equivalent values from our proposed sorting statistic for small effect sizes (*D*_s_ = 0.2), medium effect sizes (*D*_s_ = 0.45), and large effect sizes (*D*_s_ = 0.7). Note that these values are very close to the equivalent values for Cohen's *d* statistic, facilitating interpretation of the results of the proposed sorting statistic. It should also be noted that because of the sorting procedure, there is increased computational requirements for the proposed sorting statistic relative to computing Cohen's *d* statistic.

It should also be noted that the proposed sorting statistic has potential towards being applied to sortable categorical variables, a data type for which standard mathematical analysis is not normally possible. To support assessment of group-wise differences in sortable categorical variables, the equation would need to be modified to replace the difference (minus operation) with a distance metric compatible with sortable categorical variables (e.g., the number of categories separating the medians of the two distributions).
